# Regional Differences in Knee Osteoporosis Based on Coronal Alignment Phenotype in Patients Undergoing Preoperative CT Imaging

**DOI:** 10.3390/diagnostics16111747

**Published:** 2026-06-05

**Authors:** Craig E. Klinger, Maximilian M. Mueller, Robert E. Bilodeau, Joseph T. Nguyen, Jelle P. van der List, Thomas P. Sculco, Peter K. Sculco

**Affiliations:** 1 Department of Orthopaedic Surgery, Hospital for Special Surgery, New York Presbyterian Hospital, Weill Cornell Medicine, New York, NY 10021, USA; 2 Department of Trauma Surgery, Orthopaedics and Sports Traumatology, BG Klinikum Hamburg, 21033 Hamburg, Germany; 3 School of Medicine, University of Pittsburgh, Pittsburgh, PA 15261, USA; 4 Department of Orthopaedic Surgery and Sports Medicine, The Ohio State University Wexner Medical Center, Columbus, OH 43210, USA; 5 Sports Medicine Research Institute, The Ohio State University Wexner Medical Center, Columbus, OH 43210, USA

**Keywords:** osteoporosis, computed tomography, Hounsfield Units, alignment phenotype, coronal knee alignment, osteoporosis prevalence

## Abstract

**Background:** Regional periarticular bone mineral density may influence fixation and survivorship in total knee arthroplasty, but its relationship to coronal alignment remains unclear. This study assessed the association between coronal knee alignment and osteoporosis using CT Hounsfield Unit (HU)-based thresholds. **Methods:** Patients aged ≥ 50 years with standing long-leg radiographs and phantomless knee-CT (2008–2025) were retrospectively identified. Exclusion criteria included >12 months between studies, incomplete CT, non-120 kV acquisition, prior fracture or surgery, or metabolic bone disease other than osteopenia or osteoporosis. Mean trabecular attenuation was measured over 15 mm of epiphyseal bone in the distal femur and proximal tibia. Osteoporosis was defined using CT-HU thresholds. Coronal alignment was measured using hip–knee–ankle angle (HKAA) and categorized as varus (<178°), neutral (178–182°), or valgus (>182°). Measurements were performed by a reviewer blinded to osteoporosis classification. Multivariable logistic regression adjusted for age, sex, body mass index, and Kellgren–Lawrence grade. **Results:** Among 306 patients (mean age 66.9 ± 9.0 years; 51.3% female), 99.3% underwent CT for arthroplasty planning. Osteoporosis prevalence was 34.3% of varus, 58.2% of neutral, and 68.6% of valgus knees. Increasing valgus alignment was associated with higher osteoporosis odds, whereas varus alignment showed lower odds. Female sex (OR 3.06; *p* < 0.001), age (OR 1.06/year; *p* < 0.001), and HKAA (OR 1.05/degree; *p* = 0.042) remained independently associated with osteoporosis, whereas Kellgren–Lawrence grade was nonsignificant. **Conclusions:** Coronal alignment was associated with CT-defined regional knee osteoporosis. Valgus alignment showed increased odds of osteoporosis, whereas varus alignment showed lower prevalence.

## 1. Introduction

Coronal knee alignment alters tibiofemoral load distribution and may influence periarticular bone remodeling [1,2,3]. Varus alignment shifts the mechanical axis medially, increasing medial compartment loading, whereas valgus alignment increases lateral compartment loading [4,5]. Sustained mechanical loading influences trabecular remodeling, resulting in regional variation in periarticular bone mineral density [6,7].

Prior studies have demonstrated alignment-associated differences in medial and lateral compartment bone density using CT and DXA, with varus alignment associated with relatively greater medial compartment density and valgus alignment associated with lateral compartment changes [3,8,9].

Central DXA studies in patients undergoing total knee arthroplasty demonstrate substantial rates of osteoporosis; however, central measurements may not reflect local periarticular bone quality relevant to implant fixation [10,11].

Knowledge of local knee BMD by alignment phenotype may guide surgical decision-making. To our knowledge, no prior study has evaluated CT-based regional osteoporosis classification in relation to coronal alignment. The purpose of the study was to assess the association between coronal knee alignment and overall and regional knee osteoporosis using CT Hounsfield Unit-based bone mineral density thresholds. The hypothesis was that no differences in the prevalence of knee osteoporosis would be found between coronal knee alignment phenotypes.

## 2. Materials and Methods

Our Institutional Review Board approved this retrospective observational study. The study cohort was derived from an institutional imaging dataset previously assembled to evaluate relationships between lower limb alignment and regional periarticular knee bone characteristics [12]. This dataset included consecutive patients from a single academic institution between 1 January 2008 and 16 August 2025 who underwent standing anteroposterior long-leg radiographs (LLXR) and phantomless non-contrast knee CT imaging, predominantly obtained for preoperative robotic-assisted arthroplasty planning. Exclusion criteria included >12 months between imaging studies, incomplete CT coverage, non-120 kV acquisition, prior fracture or knee surgery, or metabolic bone disease other than osteopenia or osteoporosis. In contrast to the source dataset, which included adults across a broad age range, the present study restricted inclusion to individuals aged 50 years and older, as CT-based bone mineral density classification used in this analysis was derived from prior reference work in this age group [13]. All eligible cases meeting these criteria within the study timeframe were included to minimize selection bias.

### 2.1. BMD Classification

**Figure 1:**
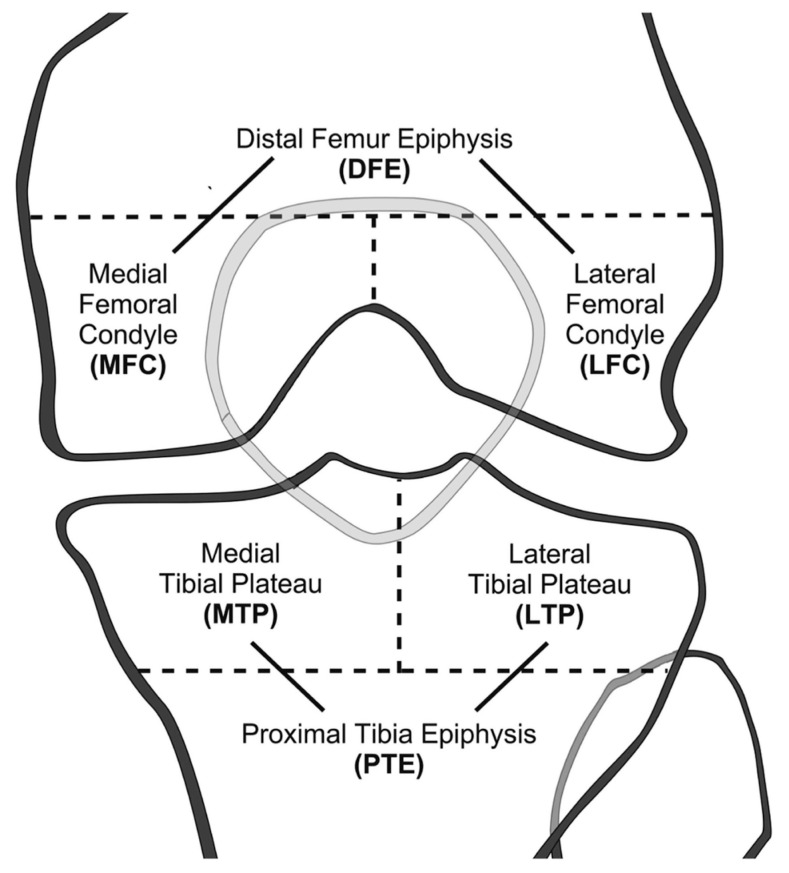
Schematic diagram of regional periarticular CT Hounsfield Unit measurement regions, including the distal femoral epiphysis (DFE), medial femoral condyle (MFC), lateral femoral condyle (LFC), proximal tibial epiphysis (PTE), medial tibial plateau (MTP), and lateral tibial plateau (LTP).

CT HU measurements were performed using a previously validated protocol [13], using an institutional picture archiving and communication system (PACS, Sectra IDS7, Linköping, Sweden). Six regions were assessed: distal femur epiphysis (DFE), medial femoral condyle (MFC), lateral femoral condyle (LFC), proximal tibia epiphysis (PTE), medial tibial plateau (MTP), and lateral tibial plateau (LTP) (Figure 1). Mean trabecular attenuation was calculated over 15 mm of epiphyseal bone while avoiding sclerotic margins. Osteoporosis was defined using established region-specific HU thresholds (DFE: ≤164, MFC: ≤137, LFC: ≤177, PTE: ≤101, MTP: ≤116, and LTP: ≤78) [13].

Coronal alignment was measured on full-length weight-bearing radiographs using the mechanical hip–knee–ankle angle (HKAA) and categorized as varus (<178°), neutral (178–182°), or valgus (>182°) [3,14]. CT HU and HKAA measurements were performed retrospectively by a reviewer blinded to osteoporosis classification outcomes. The HU measurement protocol in this study was previously assessed for intra- and inter-rater reliability, and excellent intraclass correlation coefficients were found for intra-rater (1.000) and inter-rater (0.992–1.000) reliability for all regions [13].

An institutional electronic health record (EHR) system was used to obtain demographic data. Study variables recorded included age, sex, self-reported race and ethnicity, BMI, CT scan indication and laterality, and osteoporosis status when documented.

### 2.2. Outcome Measures

Primary outcome measures included regional knee BMD classification for CT-defined regional knee osteoporosis, assessed with previously reported CT HU reference values [13], and degree of coronal deformity based on HKAA. The association of independent covariates including age, sex, and body mass index (BMI) with regional knee osteoporosis was also assessed. Regional knee HU values were separately analyzed to further evaluate the association between raw HU and coronal alignment phenotype, including a composite measurement of aggregate regional Hounsfield Units (ARHU), calculated as distal femur epiphysis HU plus proximal tibia epiphysis HU, to provide an overall assessment of epiphyseal periarticular bone density. No missing data were identified for exposure, outcome, or covariate variables. Overall knee osteoporosis, defined as osteoporosis in any assessed region, was also evaluated. This sensitive composite definition was selected to identify focal periarticular osteoporosis within any assessed compartment, given the potential local relevance of even region-specific bone deficiency to periarticular bone quality assessment.

### 2.3. Data Analysis

Analysis was performed using SPSS (v29.0.2.0; IBM, Armonk, NY, USA). Normality was assessed using the Shapiro–Wilk test. Normally distributed data are presented as mean ± standard deviation (SD), and non-normally distributed data as median and interquartile range (IQR). Associations were evaluated using Pearson’s or Spearman’s correlation coefficients, as appropriate. Group comparisons were performed using one-way ANOVA or Kruskal–Wallis tests, with Bonferroni correction for multiple comparisons. Categorical variables were compared using chi-square or Fisher’s exact tests with Holm step-down adjustment. Univariate and multivariable logistic regression analyses were conducted to assess independent associations between predictor variables and regional knee osteoporosis, adjusting for potential confounders. Multivariable models were constructed to isolate the association between coronal alignment and osteoporosis independent of demographic factors and radiographic osteoarthritis severity (Kellgren–Lawrence (KL) grade). Receiver operating characteristic (ROC) curve analysis was performed to assess model discrimination, with area under the curve (AUC) interpreted using established criteria [15,16]. ROC analysis was included as an exploratory assessment of model discrimination rather than formal validation of a clinical prediction model. Model fit was assessed using the Hosmer–Lemeshow goodness-of-fit test, and covariates were evaluated for multicollinearity. Prespecified subgroup analyses were performed stratified by sex. Sensitivity analyses included (1) categorical representation of coronal alignment (varus, neutral, valgus) in place of continuous HKAA, and (2) adjustment for radiographic osteoarthritis severity using the KL grade classification on standing LLXR. KL grade was assessed retrospectively on standing LLXR using established radiographic criteria [17,18], by a reviewer blinded to osteoporosis classification outcomes. KL grade was categorized as 0–1, 2, and 3–4 and included as a covariate in multivariable models. One knee per study patient was included in the analysis. Race and ethnicity were collected descriptively and were not incorporated into multivariable modeling due to limited subgroup sample sizes.

## 3. Results

### 3.1. Cohort Characteristics

**Figure 2:**
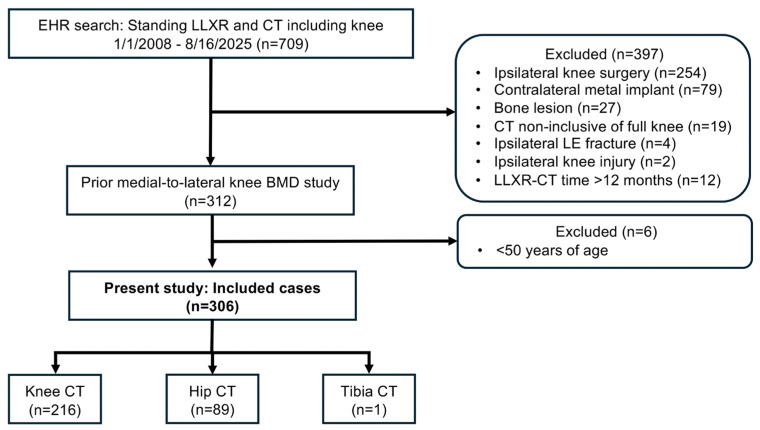
Flowchart detailing study cohort development based on eligibility criteria. CT = Computed tomography; LE = lower extremity; LLXR = long-leg x-ray; and BMD = bone mineral density.

**Figure 3:**
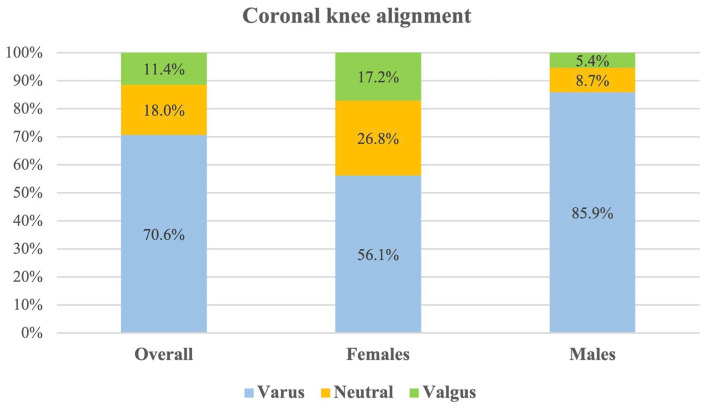
Coronal knee alignment distribution based on hip–knee–ankle angle classified as varus (<178°), neutral (178–182°), or valgus (>182°).

A total of 709 patients were screened, of whom 306 met inclusion criteria (mean age 66.9 ± 9.0 years; 51.3% female) (Figure 2, Table 1). Alignment distribution was 70.6% varus (*n* = 216), 18.0% neutral (*n* = 55), and 11.4% valgus (*n* = 35), with a mean HKAA of 175.1° ± 5.5° (range 160.2–194.3°) (Table 2; Figure 3). Mean BMI was 28.4 ± 5.3 kg/m^2^. Mean HKAA differed significantly by sex (females: 176.7°, males: 173.4°; *p* < 0.001). CT imaging was obtained for robotic-assisted arthroplasty in 99.3% of cases.

### 3.2. Overall and Regional Osteoporosis Prevalence

Overall knee osteoporosis (defined as osteoporosis in any region) was present in 42.5% of the cohort. Prevalence differed significantly by alignment phenotype: 34.3% in varus knees, 58.2% in neutral knees, and 68.6% in valgus knees (varus vs. neutral *p* = 0.003; varus vs. valgus *p* = 0.002). Across all six regions, varus knees consistently demonstrated the lowest regional osteoporosis prevalence, whereas valgus knees demonstrated the highest (Table 3, Table S1). Similar patterns were observed when stratified by sex, with significant differences among females but not males. Mean regional Hounsfield Unit values were significantly higher in varus knees compared with neutral and valgus knees across femoral and tibial regions (Tables S2 and S3). Increasing HKAA correlated with lower regional HU values, strongest in the medial tibial plateau (r = −0.372, *p* < 0.001). Age correlated negatively and BMI positively with regional HU values (Table S4).

### 3.3. Logistic Regression Analyses

In univariable analysis, increasing HKAA, female sex, and age were associated with increased odds of knee osteoporosis, while BMI was inversely associated. In multivariable analysis adjusting for age, sex, and BMI, higher HKAA remained independently associated with knee osteoporosis (OR 1.05 per degree toward valgus, 95% CI 1.00–1.11, *p* = 0.042). Female sex (OR 3.06, 95% CI 1.82–5.14, *p* < 0.001) and increasing age (OR 1.06 per year, 95% CI 1.03–1.09, *p* < 0.001) were also independent predictors, while BMI was not significant (Table S5). A 5° increase toward valgus alignment corresponded to approximately 28% increased odds of knee osteoporosis.

The continuous HKAA model demonstrated fair discrimination for overall knee osteoporosis (AUC = 0.740, *p* < 0.001). Regional AUC values ranged from 0.721 to 0.805, indicating fair-to-good performance across compartments. Similar results were observed in sensitivity analyses using categorical alignment, when coronal knee alignment was categorized as varus, neutral, valgus (Table S6). Full multivariable regression model outputs, including odds ratios, confidence intervals, and *p*-values for all regional models, are provided in Supplementary Tables S5 and S6.

In multivariable sensitivity analyses additionally adjusting for radiographic osteoarthritis severity (KL grade), coronal alignment remained significantly associated with overall knee osteoporosis after adjustment (*p* = 0.005). KL grade was not significantly associated with knee osteoporosis in the adjusted model (overall *p* = 0.093). Female sex and increasing age remained significant predictors, while BMI was not significant. Model discrimination remained similar following KL adjustment (AUC = 0.752, 95% CI 0.695–0.808).

## 4. Discussion

The principal finding of this study was that coronal alignment remained significantly associated with CT-defined regional knee osteoporosis after adjustment for demographic factors and radiographic osteoarthritis severity, with progressive valgus alignment associated with increasing odds of osteoporosis. The lowest prevalence was observed in varus knees (34.3%) and the highest in valgus knees (68.6%), followed by neutral knees (58.2%).

Prior studies have demonstrated compartment-specific bone density differences by alignment phenotype [3,8,9,19]. The present study extends these findings by suggesting that alignment is independently associated with CT-defined regional osteoporosis classification across the knee.

In multivariable logistic regression models, higher HKAA remained significantly associated with knee osteoporosis after adjustment for age, sex, and BMI, with each one-degree increase toward valgus associated with a 5% increase in odds. These findings suggest that the association between coronal alignment and osteoporosis persists after accounting for key demographic factors. This pattern was also observed in females, in whom the significant differences in overall osteoporosis prevalence persisted after stratification by sex.

The observed association between coronal alignment and regional osteoporosis persisted following adjustment for radiographic osteoarthritis severity, suggesting that the findings are not solely explained by OA severity. Although the strength of association varied across individual regions, particularly within some lateral compartment analyses, the overall pattern supports a relationship between coronal alignment phenotype and regional periarticular bone quality. Because of the retrospective cross-sectional design, causality cannot be determined, and it remains unclear whether coronal alignment contributed to regional osteoporosis development, whether bone quality influenced deformity progression, or whether both reflect osteoarthritis-related remodeling processes.

Female sex was a strong independent predictor of knee osteoporosis, with females demonstrating over threefold increased odds compared with males in multivariable analysis. These findings align with prior reports of sex-based differences in skeletal fragility patterns [20,21]. The observed sex-specific differences in regional knee bone density underscore the relevance of local periarticular bone assessment and may have implications for surgical planning, particularly in patients considered for cementless fixation strategies.

Increasing age independently predicted knee osteoporosis, with each additional year associated with approximately 6% increased odds in multivariable analysis. This finding is consistent with prior literature demonstrating age-related increases in osteoporosis risk in the central skeleton [22,23,24,25,26].

These findings may have potential implications for future investigations of TKA planning and fixation strategy selection, although the present study did not evaluate surgical outcomes, implant migration, or fixation performance directly. Accordingly, these findings should be considered hypothesis-generating rather than directly practice-changing. Prospective studies evaluating the relationship between regional periarticular bone quality, fixation strategy selection, and postoperative arthroplasty outcomes are warranted. Assessment of local knee BMD is of clinical importance, as research in TKA patients, has found an association between early postoperative component migration in uncemented TKA and mean BMD [19]. Andersen et al., in a study on 92 patients with uncemented tibial TKA components who underwent preoperative DXA, found low preoperative BMD significantly associated with higher maximum total point motion at all follow-up timepoints (3, 6, 12, 24 months) [27].

Despite these potentially relevant clinical associations, the present findings should be interpreted within the context of important study limitations.

This study had several limitations. As the study sample consisted of patients 50 years of age and older who were primarily indicated for hip or knee arthroplasty procedures, osteoarthritis was common, which may limit generalizability. Although subchondral sclerosis associated with OA can influence local HU measurements, regional CT measurements were performed with the aim of avoiding visible hyperdense sclerotic margins, and mean HU values were obtained across 15 mm of trabecular bone to reduce focal measurement bias. Importantly, in multivariable sensitivity analyses adjusting for radiographic osteoarthritis severity, KL grade was not independently associated with knee osteoporosis, and the association between coronal alignment and osteoporosis persisted, suggesting that the observed association between coronal alignment and regional knee osteoporosis is independent of radiographic OA severity. Additionally, compartment-specific osteoarthritis severity was not separately analyzed and may represent a more localized determinant of periarticular remodeling than global KL grade. As the study primarily consisted of a hip and knee arthroplasty population, the observed osteoporosis prevalence and alignment distributions may not reflect those of the general population. Alternative explanations should also be considered when interpreting these findings. Prior work has demonstrated that varus and valgus knees may exhibit distinct underlying bone morphotypes and constitutional skeletal differences that may exist independent of osteoarthritis progression [28]. Accordingly, the observed regional CT-based bone density differences may reflect a combination of developmental skeletal variation, constitutional morphologic phenotype, and mechanical loading adaptation rather than loading-related remodeling alone. Alternative composite definitions, including multi-region or compartment-specific classifications, may yield different prevalence estimates and should be explored in future studies. Furthermore, osteoporosis treatment status was not incorporated into multivariable modeling, and anti-osteoporotic therapies may influence regional HU measurements. Lastly, a limited number of valgus cases were identified (35 overall; 27 females and 8 males), which may have limited power to detect differences within the male cohort. Accordingly, nonsignificant findings in male subgroup analyses should not be interpreted as evidence of absence of association. However, all eligible cases were included to minimize selection bias.

This study also had several important strengths. It is the first to assess CT-based regional knee osteoporosis classification in relation to coronal alignment phenotype, providing novel observational data. The relatively large cohort allowed evaluation of independent associations between alignment, demographics, and regional BMD. Varus knees demonstrated the lowest overall osteoporosis prevalence across the entire knee.

## 5. Conclusions

Coronal alignment was significantly associated with CT-defined regional knee osteoporosis after adjustment for demographic factors and radiographic osteoarthritis severity. Progressive valgus alignment was associated with increased odds of osteoporosis, whereas varus alignment demonstrated lower prevalence. These findings suggest that coronal alignment phenotype is associated with regional differences in periarticular bone quality independent of radiographic osteoarthritis severity.

## Figures and Tables

**Table 1:** Baseline characteristics and radiographic details.

Variables (*N* = 306)		Mean (SD)	Range	*N* (%)
Age		66.9 (9.0)	50–91	306 (100.0)
	Female	67.3 (8.8)	51–88	157 (51.3)
	Male	66.5 (9.2)	50–91	149 (48.7)
Body Mass Index (BMI)		28.4 (5.3)	16.4–48.0	306 (100.0)
	Underweight (<18.5)			2 (0.7)
	Normal (18.5–24.9)			81 (26.5)
	Overweight (25–29.9)			108 (35.3)
	Obese (30–40)			107 (35.0)
	Severely obese (>40)			8 (2.6)
Race	White			269 (87.9)
	Unknown			19 (6.2)
	Black or African American			11 (3.6)
	Asian			6 (2.0)
	American Indian or Alaska Native			1 (0.3)
Ethnicity	Not Hispanic or Latino			285 (93.1)
	Hispanic or Latino			15 (4.9)
	Unknown			6 (2.0)
CT indication	Lower limb alignment assessment			2 (0.7)
	Robotic-assisted THA, TKA, or UKA			304 (99.3)
	Total hip arthroplasty			88 (28.8)
	Total knee arthroplasty			39 (12.7)
	Unicondylar knee arthroplasty			176 (57.5)
	Patellofemoral arthroplasty			1 (0.3)
KL grade	0–1			98 (32.0)
	2			83 (27.1)
	3–4			125 (40.8)
Knee measured	Right			147 (48.0)
	Left			159 (52.0)
Days between standing LLXR and CT		15.7 (39.3)	0.0–273.0	306 (100.0)
Undergoing osteoporosis treatment				
	Yes			35 (11.4)
	No			271 (88.6)

THA = total hip arthroplasty, TKA = total knee arthroplasty, and UKA = unicompartmental knee arthroplasty. KL = Kellgren–Lawrence. Race and ethnicity data are self-reported.

**Table 2:** Knee alignment groups based on hip–knee–ankle angle.

HKAA Group		Boundaries	*N* (%)	Mean (SD)
**Study** **cohort (** ***n*** ** = 306)**				
**Overall**		**NA**	**306 (100)**	**175.1° (5.5°)**
	Varus	<178°	216 (70.6)	172.3° (3.5°)
	Neutral	178–182°	55 (18.0)	179.5° (1.1°)
	Valgus	>182°	35 (11.4)	185.2° (2.7°)
**Females (** ***n*** ** = 157)**				
		**NA**	**157 (100)**	**176.7° (5.4°)**
	Varus	<178°	88 (56.1)	172.9° (3.4°)
	Neutral	178–182°	42 (26.8)	179.6° (1.2°)
	Valgus	>182°	27 (17.2)	184.7° (2.3°)
**Males (** ***n*** ** = 149)**				
		**NA**	**149 (100)**	**173.4° (5.1°)**
	Varus	<178°	128 (85.9)	171.9° (3.6°)
	Neutral	178–182°	13 (8.7)	179.1° (0.7°)
	Valgus	>182°	8 (5.4)	186.9° (3.4°)

HKAA = hip–knee–ankle angle, SD = standard deviation, NA = not applicable.

**Table 3:** Osteoporosis prevalence by alignment phenotype and by sex.

Results		Category	Overall *N* (%AP)	Females *N* (%AP)	Males *N* (%AP)
**Overall** osteoporosis prevalence *		Overall	130 (42.5)	90 (57.3)	40 (26.8)
		Neutral (178–182°)	32 (58.2)	29 (69.0)	3 (23.1)
		Varus (<178°)	74 (34.3)	41 (46.6)	33 (25.8)
		Valgus (>182°)	24 (68.6)	20 (74.1)	4 (50.0)
**Osteoporosis prevalence by region**					
	Distal femur epiphysis (DFE)	Overall	110 (35.9)	81 (51.6)	29 (19.5)
		Neutral (178–182°)	27 (49.1)	26 (61.9)	1 (7.7)
		Varus (<178°)	60 (27.8)	36 (40.9)	24 (18.8)
		Valgus (>182°)	23 (65.7)	19 (70.4)	4 (50.0)
	Medial femoral condyle (MFC)	Overall	92 (30.1)	71 (45.2)	21 (14.1)
		Neutral (178–182°)	27 (49.1)	25 (59.5)	2 (15.4)
		Varus (<178°)	43 (19.9)	28 (39.8)	15 (11.7)
		Valgus (>182°)	22 (62.9)	18 (66.7)	4 (50.0)
	Lateral femoral condyle (LFC)	Overall	111 (36.3)	80 (51.0)	31 (20.8)
		Neutral (178–182°)	28 (50.9)	27 (64.3)	1 (7.7)
		Varus (<178°)	66 (30.6)	38 (43.2)	28 (21.9)
		Valgus (>182°)	17 (48.6)	15 (55.6)	2 (25.0)
	Proximal tibia epiphysis (PTE)	Overall	82 (26.8)	65 (41.4)	17 (11.4)
		Neutral (178–182°)	23 (41.8)	21 (50.0)	2 (15.4)
		Varus (<178°)	41 (19.0)	28 (31.8)	13 (10.2)
		Valgus (>182°)	18 (51.4)	16 (59.3)	2 (25.0)
	Medial tibial plateau (MTP)	Overall	74 (24.2)	56 (35.7)	18 (12.1)
		Neutral (178–182°)	24 (43.6)	22 (52.4)	2 (15.4)
		Varus (<178°)	30 (13.9)	18 (20.5)	12 (9.4)
		Valgus (>182°)	20 (57.1)	16 (59.3)	4 (50.0)
	Lateral tibial plateau (LTP)	Overall	72 (23.5)	57 (36.3)	15 (10.1)
		Neutral (178–182°)	20 (36.4)	19 (45.2)	1 (7.7)
		Varus (<178°)	43 (19.9)	31 (35.2)	12 (9.4)
		Valgus (>182°)	9 (25.7)	7 (25.9)	2 (25.0)

%AP = percentage of each alignment phenotype group. * Overall osteoporosis prevalence is based on the presence of osteoporosis in any region. BMD classification based on most severe classification within each specified knee region.

## Data Availability

The data presented in this study are not publicly available due to privacy and institutional restrictions, including the presence of protected health information (PHI). Data may be available from the corresponding author upon reasonable request and with appropriate institutional approvals.

## Supplementary Materials

The following supporting information can be downloaded at: https://www.mdpi.com/article/10.3390/diagnostics16111747/s1, Table S1: Pairwise analysis for knee osteoporosis prevalence; Table S2: Overall regional mean Hounsfield Units by alignment category; Table S3: Overall pairwise analysis of mean CT HU by alignment category; Table S4: Correlation coefficients between CT HU and clinical variables; Table S5: Binary logistic regression predicting osteoporosis; Table S6: Binary logistic regression predicting osteoporosis (categorical HKAA).

## Abbreviations

The following abbreviations are used in this manuscript:

ARHU	Aggregate Regional Hounsfield Units
AUC	Area Under the Curve
BMI	Body Mass Index
BMD	Bone Mineral Density
CT	Computed Tomography
DFE	Distal Femur Epiphysis
DXA	Dual-Energy X-ray Absorptiometry
EHR	Electronic Health Record
HKAA	Hip–Knee–Ankle Angle
HU	Hounsfield Units
IQR	Interquartile Range
KL	Kellgren–Lawrence
LLXR	Long-Leg X-ray
LFC	Lateral Femoral Condyle
LTP	Lateral Tibial Plateau
MFC	Medial Femoral Condyle
MTP	Medial Tibial Plateau
PACS	Picture Archiving and Communication System
PTE	Proximal Tibia Epiphysis
ROC	Receiver Operating Characteristic
SD	Standard Deviation
TKA	Total Knee Arthroplasty
