# Supplementary material for: Regional Differences in Knee Osteoporosis Based on Coronal Alignment Phenotype in Patients Undergoing Preoperative CT Imaging
Source: Diagnostics (Basel). 2026 Jun 5;16(11):1747. doi: 10.3390/diagnostics16111747 (PMC13256476; doi:10.3390/diagnostics16111747)
Supplement: Supplementary file 1 [file diagnostics-16-01747-s001.zip › Table S1.pdf]

Table S1. Pairwise analysis for knee osteoporosis prevalence.

Pairwise comparison		Category	Overall p-value	Females p-value	Males p-value
**Osteoporosis**	Osteoporosis in any region	Varus - neutral	**0.003**†	**0.032**†	>0.999‡
		Varus - valgus	**0.002**†	**0.036**†	0.612‡
		Valgus - neutral	0.322†	0.653†	0.612‡
	Distal femur epiphysis (DFE)	Varus - neutral	**0.006**†	**0.050**†	0.464‡
		Varus - valgus	**0.003**†	**0.021**†	0.141‡
		Valgus - neutral	0.122†	0.471†	0.141‡
	Medial femoral condyle (MFC)	Varus - neutral	**0.003**†	**0.006**†	0.658‡
		Varus - valgus	**0.002**†	**0.003**†	**0.042**‡
		Valgus - neutral	0.201†	0.550†	0.292‡
	Lateral femoral condyle (LFC)	Varus - neutral	**0.015**†	0.072†	0.912‡
		Varus - valgus	0.072†	0.518†	>0.999‡
		Valgus - neutral	0.829†	0.468†	>0.999‡
	Proximal tibia epiphysis (PTE)	Varus - neutral	**0.003**†	0.090†	>0.999‡
		Varus - valgus	**0.003**†	**0.030**†	0.645‡
		Valgus - neutral	0.372†	0.452†	>0.999‡
	Medial tibial plateau (MTP)	Varus - neutral	**0.003**†	**0.003**†	0.619‡
		Varus - valgus	**0.003**†	**0.002**†	**0.021**‡
		Valgus - neutral	0.211†	0.575†	0.292‡
	Lateral tibial plateau (LTP)	Varus - neutral	**0.030**†	0.546†	>0.999‡
		Varus - valgus	0.432†	0.369†	0.576‡
		Valgus - neutral	0.584†	0.318†	>0.999‡

†Chi-square test; Holm-corrected, ‡Fisher's exact test; Holm-corrected.
