# Supplementary material for: Regional Differences in Knee Osteoporosis Based on Coronal Alignment Phenotype in Patients Undergoing Preoperative CT Imaging
Source: Diagnostics (Basel). 2026 Jun 5;16(11):1747. doi: 10.3390/diagnostics16111747 (PMC13256476; doi:10.3390/diagnostics16111747)
Supplement: Supplementary file 1 [file diagnostics-16-01747-s001.zip › Table S2.pdf]

Table S2. Overall regional mean Hounsfield Units by alignment category.

**Results (N=306)**	**Category**	**HU Mean (SD)**	**HU Range**
Aggregate regional HU (ARHU)	Neutral (178–182°)	285.6 (108.8)	76.6-559.7
	Varus (<178°)	345.5 (42.4)	42.4-615.3
	Valgus (>182°)	277.2 (111.7)	52.4-551.0
Distal femur epiphysis (DFE)	Neutral (178–182°)	167.9 (62.9)	47.2-324.3
	Varus (<178°)	200.8 (56.3)	35.8-346.3
	Valgus (>182°)	161.7 (58.8)	40.5-319.3
Medial femoral condyle (MFC)	Neutral (178–182°)	148.2 (61.1)	34.8-300.0
	Varus (<178°)	186.3 (55.0)	27.2-315.3
	Valgus (>182°)	132.2 (61.5)	6.2-296.3
Lateral femoral condyle (LFC)	Neutral (178–182°)	186.0 (66.2)	46.4-347.3
	Varus (<178°)	214.4 (60.5)	43.6-377.7
	Valgus (>182°)	189.0 (57.7)	72.7-340.0
Proximal tibia epiphysis (PTE)	Neutral (178–182°)	117.7 (47.7)	29.5-235.3
	Varus (<178°)	144.7 (46.8)	6.5-283.7
	Valgus (>182°)	115.4 (54.3)	11.9-260.3
Medial tibial plateau (MTP)	Neutral (178–182°)	131.7 (49.3)	31.6-250.3
	Varus (<178°)	166.9 (47.5)	11.6-291.3
	Valgus (>182°)	117.2 (61.8)	-9.5-273.0
Lateral tibial plateau (LTP)	Neutral (178–182°)	103.7 (47.8)	27.7-222.0
	Varus (<178°)	123.0 (49.3)	1.8-276.0
	Valgus (>182°)	113.8 (49.7)	36.1-248.0

ARHU=Aggregate regional Hounsfield Units (DFE HU+PTE HU).
