# Supplementary material for: Regional Differences in Knee Osteoporosis Based on Coronal Alignment Phenotype in Patients Undergoing Preoperative CT Imaging
Source: Diagnostics (Basel). 2026 Jun 5;16(11):1747. doi: 10.3390/diagnostics16111747 (PMC13256476; doi:10.3390/diagnostics16111747)
Supplement: Supplementary file 1 [file diagnostics-16-01747-s001.zip › Table S3.pdf]

Table S3. Overall pairwise analysis of mean CT HU by alignment category.

Pairwise Comparison	Category	Overall p-value
**Comparisons by Hounsfield Units**		
Aggregate regional Hounsfield Units (ARHU)	Varus-neutral	**0.001†**
	Varus-valgus	**0.001†**
	Valgus-neutral	>0.999†
Distal femur epiphysis (DFE)	Varus-neutral	**0.001†**
	Varus-valgus	**0.001†**
	Valgus-neutral	>0.999†
Medial femoral condyle (MFC)	Varus-neutral	**<0.001†**
	Varus-valgus	**<0.001†**
	Valgus-neutral	0.691†
Lateral femoral condyle (LFC)	Varus-neutral	**0.006†**
	Varus-valgus	**<0.040†**
	Valgus-neutral	>0.999†
Proximal tibia epiphysis (PTE)	Varus-neutral	**0.001†**
	Varus-valgus	**0.001†**
	Valgus-neutral	>0.999†
Medial tibial plateau (MTP)	Varus-neutral	**<0.001†**
	Varus-valgus	**<0.001†**
	Valgus-neutral	0.855†
Lateral tibial plateau (LTP)	Varus-neutral	**0.018†**
	Varus-valgus	0.608†
	Valgus-neutral	>0.999†

†Kruskal-Wallis Independent-Samples test; significance adjusted by Bonferroni correction. HU=Hounsfield Units.
