# Supplementary material for: Regional Differences in Knee Osteoporosis Based on Coronal Alignment Phenotype in Patients Undergoing Preoperative CT Imaging
Source: Diagnostics (Basel). 2026 Jun 5;16(11):1747. doi: 10.3390/diagnostics16111747 (PMC13256476; doi:10.3390/diagnostics16111747)
Supplement: Supplementary file 1 [file diagnostics-16-01747-s001.zip › Table S4.pdf]

Table S4. Correlation coefficients between CT HU and clinical variables.

	Correlation Coefficients†		
**Region**	**HKAA**	**Age (Sig.)**	**BMI (Sig.)**
**Overall (n=306)**			
ARHU	-0.250 **(p<0.001)**	-0.212 **(p<0.001)**	0.240 **(p<0.001)**
Distal femur epiphysis	-0.247 **(p<0.001)**	-0.185 **(p=0.001)**	0.219 **(p<0.001)**
Medial femoral condyle	-0.323 **(p<0.001)**	-0.229 **(p<0.001)**	0.232 **(p<0.001)**
Lateral femoral condyle	-0.168 **(p=0.003)**	-0.135 **(p=0.018)**	0.201 **(p<0.001)**
Proximal tibia epiphysis	-0.242 **(p<0.001)**	-0.235 **(p<0.001)**	0.254 **(p<0.001)**
Medial tibial plateau	-0.372 (**p<0.001**)	-0.202 **(p<0.001)**	0.276 **(p<0.001)**
Lateral tibial plateau	-0.094 (p=0.100)	-0.235 **(p<0.001)**	0.207 **(p<0.001)**
**Females (n=157)**			
ARHU	-0.240 **(p=0.002)**	-0.198 **(p=0.013)**	0.177 **(p=0.027)**
Distal femur epiphysis	-0.237 **(p=0.003)**	-0.184 **(p=0.021)**	0.152 (p<0.057)
Medial femoral condyle	-0.357 **(p<0.001)**	-0.208 **(p=0.009)**	0.166 **(p=0.038)**
Lateral femoral condyle	-0.117 (p=0.146)	-0.154 (p=0.055)	0.135 (p=0.093)
Proximal tibia epiphysis	-0.233 **(p=0.003)**	-0.207 **(p<0.009)**	0.199 **(p=0.012)**
Medial tibial plateau	-0.393 (**p<0.001**)	-0.155 (p=0.052)	0.224 **(p=0.005)**
Lateral tibial plateau	-0.030 (p=0.711)	-0.237 **(p=0.003)**	0.144 **(p=0.072)**
**Males (n=149)**			
ARHU	-0.031 (p=0.706)	-0.230 **(p=0.005)**	0.184 **(p=0.025)**
Distal femur epiphysis	-0.023 (p=0.783)	-0.183 **(p=0.025)**	0.160 (p=0.051)
Medial femoral condyle	-0.088 (p=0.285)	-0.255 **(p=0.002)**	0.186 **(p=0.023)**
Lateral femoral condyle	0.041 (p=0.616)	-0.104 (p=0.207)	0.132 (p=0.108)
Proximal tibia epiphysis	-0.039 (p=0.640)	-0.267 (**p<0.001**)	0.198 **(p=0.016)**
Medial tibial plateau	-0.186 (**p=0.023**)	-0.249 (**p=0.002**)	0.236 **(p=0.004)**
Lateral tibial plateau	0.103 (p=0.211)	-0.257 **(p=0.002)**	0.143 (p=0.082)

†Pearson correlation coefficient, HU=Hounsfield Units.

ARHU=Aggregate regional Hounsfield Units (DFE HU+PTE HU), Sig.=p-value.
