# Supplementary material for: Regional Differences in Knee Osteoporosis Based on Coronal Alignment Phenotype in Patients Undergoing Preoperative CT Imaging
Source: Diagnostics (Basel). 2026 Jun 5;16(11):1747. doi: 10.3390/diagnostics16111747 (PMC13256476; doi:10.3390/diagnostics16111747)
Supplement: Supplementary file 1 [file diagnostics-16-01747-s001.zip › Table S5.pdf]

Table S5. Binary logistic regression predicting osteoporosis.

Predictor	OR	95% CI	p-value
**Overall osteoporosis status**			
Female (reference: Male)	3.061	1.822-5.142	**<0.001**
Age (years)	1.056	1.026-1.086	**<0.001**
HKAA (degrees)	1.053	1.002-1.106	**0.042**
BMI (kg/m^2)	0.969	0.922-1.018	0.215
**DFE osteoporosis status**			
Female (reference: Male)	3.598	2.089-6.196	**<0.001**
Age (years)	1.042	1.012-1.072	**0.006**
HKAA (degrees)	1.059	1.007-1.113	**0.024**
BMI (kg/m^2)	0.970	0.922-1.021	0.241
**MFC osteoporosis status**			
Female (reference: Male)	3.688	1.994-6.820	**<0.001**
Age (years)	1.064	1.029-1.099	**<0.001**
HKAA (degrees)	1.135	1.072-1.202	**<0.001**
BMI (kg/m^2)	0.971	0.919-1.027	0.310
**LFC osteoporosis status**			
Female (reference: Male)	3.630	2.129-6.191	**<0.001**
Age (years)	1.032	1.004-1.062	**0.025**
HKAA (degrees)	1.010	0.963-1.060	0.676
BMI (kg/m^2)	0.969	0.923-1.019	0.219
**PTE osteoporosis status**			
Female (reference: Male)	4.171	2.219-7.842	**<0.001**
Age (years)	1.038	1.006-1.071	**0.020**
HKAA (degrees)	1.095	1.037-1.156	**0.001**
BMI (kg/m^2)	0.975	0.923-1.031	0.374
**MTP osteoporosis status**			
Female (reference: Male)	2.494	1.285-4.839	**0.007**
Age (years)	1.040	1.005-1.076	**0.023**
HKAA (degrees)	1.188	1.115-1.265	**<0.001**
BMI (kg/m^2)	0.972	0.915-1.033	0.360
**LTP osteoporosis status**			
Female (reference: Male)	4.810	2.496-9.272	**<0.001**
Age (years)	1.046	1.014-1.080	**0.005**
HKAA (degrees)	1.019	0.965-1.075	0.498
BMI (kg/m^2)	0.992	0.940-1.048	0.785

OR=odds ratio.

Models adjusted for sex, age, BMI, and HKAA (continuous variable).
