# Supplementary material for: Regional Differences in Knee Osteoporosis Based on Coronal Alignment Phenotype in Patients Undergoing Preoperative CT Imaging
Source: Diagnostics (Basel). 2026 Jun 5;16(11):1747. doi: 10.3390/diagnostics16111747 (PMC13256476; doi:10.3390/diagnostics16111747)
Supplement: Supplementary file 1 [file diagnostics-16-01747-s001.zip › Table S6.pdf]

Table S6. Binary logistic regression predicting osteoporosis (categorical HKAA).

Predictor	OR	95% CI	p-value
**Overall osteoporosis status**			
Female (reference: Male)	2.896	1.717-4.883	**<0.001**
Age (years)	1.055	1.025-1.085	**<0.001**
HKAA - Varus (vs Neutral)	0.525	0.265-1.042	0.065
HKAA - Valgus (vs Neutral)	1.479	0.570-3.839	0.421
BMI (kg/m²)	0.970	0.923-1.020	0.233
**DFE osteoporosis status**			
Female (reference: Male)	3.482	2.010-6.032	**<0.001**
Age (years)	1.039	1.010-1.070	**0.009**
HKAA - Varus (vs Neutral)	0.623	0.314-1.238	0.177
HKAA - Valgus (vs Neutral)	2.014	0.790-5.134	0.143
BMI (kg/m²)	0.967	0.919-1.018	0.203
**MFC osteoporosis status**			
Female (reference: Male)	3.621	2.013-6.512	**<0.001**
Age (years)	1.059	1.025-1.094	**0.001**
HKAA - Varus (vs Neutral)	0.373	0.181-0.769	**0.007**
HKAA - Valgus (vs Neutral)	1.712	0.641-4.573	0.283
BMI (kg/m²)	0.963	0.906-1.023	0.223
**LFC osteoporosis status**			
Female (reference: Male)	3.419	2.034-5.747	**<0.001**
Age (years)	1.034	1.005-1.063	**0.021**
HKAA - Varus (vs Neutral)	0.653	0.352-1.212	0.176
HKAA - Valgus (vs Neutral)	0.846	0.408-1.756	0.651
BMI (kg/m²)	0.975	0.932-1.021	0.283
**PTE osteoporosis status**			
Female (reference: Male)	4.191	2.271-7.734	**<0.001**
Age (years)	1.036	1.005-1.067	**0.021**
HKAA - Varus (vs Neutral)	0.527	0.268-1.037	0.063
HKAA - Valgus (vs Neutral)	1.461	0.564-3.785	0.429
BMI (kg/m²)	0.968	0.917-1.022	0.235
**MTP osteoporosis status**			
Female (reference: Male)	2.499	1.307-4.780	**0.006**
Age (years)	1.037	1.004-1.071	**0.027**
HKAA - Varus (vs Neutral)	0.293	0.138-0.620	**0.001**
HKAA - Valgus (vs Neutral)	1.713	0.675-4.345	0.256
BMI (kg/m²)	0.962	0.907-1.020	0.187
**LTP osteoporosis status**			
Female (reference: Male)	4.890	2.544-9.409	**<0.001**
Age (years)	1.048	1.015-1.083	**0.004**
HKAA - Varus (vs Neutral)	0.668	0.317-1.406	0.285
HKAA - Valgus (vs Neutral)	0.509	0.204-1.270	0.148
BMI (kg/m²)	0.994	0.940-1.050	0.824

OR=odds ratio, Neutral served as reference category for categorical HKAA models.
